# Spatial distribution and determinants of missing essential newborn care items after birth in Somaliland: a spatial and multilevel analysis of SDHS

**DOI:** 10.3389/fped.2026.1732341

**Published:** 2026-02-06

**Authors:** Hana Mahdi Dahir, Ayan Husein Korse, Farduus Ibraahim Mohamed

**Affiliations:** 1 School of Postgraduate Studies and Research (SPGSR), Amoud University, Borama, Somaliland; 2 School of Postgraduate Studies and Research, University of Hargeisa, Hargeisa, Somalia

**Keywords:** determinants, DHS, essential newborn care, multilevel analysis, neonatal health, Somaliland, spatial distribution

## Abstract

**Background:**

Neonatal mortality remains a critical global health issue, particularly in low- and middle-income countries, with suboptimal essential newborn care (ENC) practices contributing to poor outcomes. This study addresses the limited evidence on spatial variations and determinants of these gaps in contexts like Somaliland.

**Methods:**

This study employed a community-based cross-sectional study design using data from the 2020 Somaliland Demographic and Health Survey (SDHS). A weighted sample of 2,314 mothers who gave birth in the five years preceding the survey was included. We performed spatial and multilevel analyses to identify determinants of missing essential newborn care. Multilevel logistic regression was used to identify individual and community-level factors, while Moran's I and Getis-Ord Gi statistics were applied to assess spatial autocorrelation and clustering.

**Results:**

A high prevalence of missing essential newborn care (87%) was identified. Spatial analysis using Global Moran's I revealed significant positive spatial autocorrelation, indicating non-random clustering of missing care. Getis-Ord Gi statistics identified significant hot spots (areas with high missing care) in the eastern regions (Sanaag, Sool, and Togdheer) and cold spots (low missing care) in the western regions (Awdal and Marodijeh). Multilevel analysis revealed that delivery in a health facility dramatically increased the likelihood of receiving ENC (AOR = 6.17; 95% CI: 4.24–8.97) compared to home births. Mothers from middle (AOR = 2.16) and rich (AOR = 2.28) wealth quintiles were more likely to receive ENC, and children of average size were less likely to miss care (AOR = 0.46; 95% CI: 0.30–0.71).

**Conclusion:**

This study highlights a substantial unmet need for essential newborn care in Somaliland, driven by significant spatial disparities and strong associations with place of delivery and socioeconomic status. These findings call for targeted interventions promoting facility-based deliveries, addressing wealth-based inequalities, and strengthening regional health systems to enhance ENC coverage and improve neonatal survival.

## Introduction

Neonatal mortality, defined as the death of a newborn within the first 28 days of life, remains a pressing global health challenge. Over 98% of neonatal deaths occur in low- and middle-income countries (LMICs), underscoring the urgent need for effective postnatal care interventions to improve survival outcomes (1, 2). The postnatal period, extending from birth up to six weeks, is a critical window for preventing maternal and newborn complications that can be fatal if unaddressed (3). Despite the availability of evidence-based practices, adherence to essential newborn care remains suboptimal in many settings. For instance, studies show that only 38.4% of mothers in Ethiopia demonstrate comprehensive newborn care practices, and postnatal care coverage consistently lags behind antenatal care globally (4).

Evaluating effective coverage for maternal and newborn health interventions is challenging due to significant heterogeneity in definitions and measurement methods, which hampers comparability across different contexts and time periods (5). To address these issues, the World Health Organization (WHO) recommends a minimum of four postnatal care contacts, emphasizing the first two weeks after birth as a vital period for health promotion, early problem detection, and supporting the transition to ongoing maternal and child health services (3). Within this critical framework, neonatal intensive care nurses play a pivotal role in delivering essential care activities, including communication and support for parents, which significantly influence parent satisfaction and the long-term health and developmental outcomes of infants (6).

The quality of postnatal care delivery is further compromised by significant “know-do” gaps, where healthcare providers possess adequate knowledge but struggle to translate it into consistent practice. A cross-sectional study in rural Uganda, for example, revealed substantial disparities between provider knowledge (70%) and actual quality of care delivery (45%), resulting in a mean know-do gap of 25%. The largest gaps were observed in critical areas such as infection control, vital signs monitoring, and prevention of postpartum hemorrhage (7). These implementation failures are compounded by systemic challenges within neonatal intensive care units (NICUs), where “missed nursing care”—defined as essential care activities that are omitted or delayed—significantly impacts patient outcomes. Studies have documented the widespread occurrence of missed care, with over 75% of nurses reporting occasional delays in timely documentation of maternal-fetal assessments and monitoring of intake and output. These delays are primarily attributed to inadequate staffing, resource constraints, and communication breakdowns (8).

Geographic inequities further exacerbate these challenges. In Ethiopia, for instance, essential newborn care practices such as delayed bathing, proper cord care, and timely immunizations show significant regional variations, with higher coverage rates in central and northern regions compared to other areas (9). While the integration of technology-based interventions, such as electronic reminders via mobile platforms, has shown promise in reducing missed nursing care in NICU settings, sustained implementation remains challenging (10). These multifaceted barriers collectively underscore the complexity of translating evidence-based practices into consistent, high-quality postnatal care across diverse healthcare settings. This situation highlights a critical need for in-depth investigation into the spatial distribution and determinants of missing essential newborn care items, particularly in regions like Malawi, where neonatal mortality rates, though declining, remain a concern. it highlights critical gaps in equipment and supplies for intrapartum and immediate postpartum care, crucial for reducing the burden of fetal and neonatal death (11). Rwanda faces a neonatal mortality rate of 20/1,000 live births, with a national goal of reducing it to 12 deaths per 1,000 live births by 2030. This study assesses postnatal mothers’ knowledge and practice of Essential Newborn Care (ENC) in Kayonza District, Rwanda, identifying gaps in areas like breastfeeding, cord care, and thermoregulation that hinder progress towards improved newborn health outcomes (12).

Study assess the spatial distribution and determinants of missing essential newborn care items after birth in rural Southern Ethiopia, where despite national initiatives, only one-third of women and their newborns received the full Community-Based Essential Newborn Care program packages (13). Another study investigates the availability and quality of essential newborn care and neonatal resuscitation practices in public health facilities across Afghanistan, revealing significant gaps in adherence to global guidelines and a need for improved training and resources to reduce the country's high newborn mortality rate of 40 per 1,000 live births (14). Study in Burkina Faso and Côte d'Ivoire, revealing that a significant proportion of women (over 75%) did not receive vital interventions such as skin-to-skin contact and early breastfeeding initiation, highlighting the urgent need for quality improvement interventions in these regions (15). A study in Somaliland revealed that poverty significantly impedes maternal healthcare utilization, leading to lower antenatal care attendance and a preference for home births over facility deliveries, despite initiatives to provide free services (16). The neonatal mortality rate (NMR) is the number of deaths of babies under 28 days of age per 1,000 live births (17). In Somaliland, it remains alarmingly high, estimated at 42 deaths per 1,000 live births according to the 2020 Somaliland Health and Demographic Survey. According to the 2020 Somaliland Health and Demographic Survey, only one-third of births occurred in health facilities, while 67% were delivered at home, often without skilled attendance. Postnatal care coverage was critically low, with nearly 80% of mothers and newborns receiving no check-up within the first two days of life. Although breastfeeding initiation is common, only 69% of newborns were put to the breast within the first hour after birth, and other essential care practices, including cord care and thermal protection, remain inconsistent. These gaps contribute to persistently high neonatal risks and underscore the need for targeted strategies to strengthen facility-based deliveries, skilled birth attendance, and early postnatal care services in Somaliland (18).

This situation highlights a critical need for in-depth investigation into the spatial distribution and determinants of missing essential newborn care items, particularly in regions like Somaliland, where systemic barriers and geographic disparities hinder progress toward improved neonatal survival.

## Study novelty and contribution

Although spatial and multilevel analyses using Demographic and Health Survey (DHS) data have been increasingly applied to examine essential newborn care (ENC) in low- and middle-income countries, evidence from Somaliland remains extremely limited. To our knowledge, this study is the first to jointly apply spatial autocorrelation techniques and multilevel modeling to investigate the distribution and determinants of missing essential newborn care items in the Somaliland context using nationally representative data.

Unlike previous studies that relied primarily on national or regional averages, this analysis integrates spatial clustering methods with multilevel regression to identify geographically concentrated gaps in ENC coverage while simultaneously accounting for individual- and community-level influences. By explicitly mapping hotspots and cold spots of missing ENC and linking them to socioeconomic and health service factors, this study provides location-specific evidence that is directly relevant for health system planning in Somaliland.

In a fragile and resource-constrained health system characterized by high rates of home delivery, nomadic populations, and regional inequities in service availability, this combined spatial–multilevel approach offers new insights into where and why newborn care gaps persist. The findings therefore move beyond descriptive reporting to generate actionable evidence for geographically targeted and context-specific maternal and newborn health interventions in Somaliland.

## Methods

### Study area

The study was conducted in Somaliland, a self-declared state in the Horn of Africa. Somaliland is divided into six administrative regions: Awdal, Marodijeh, Sahil, Togdheer, Sool, and Sanaag. According to the 2020 Somaliland Health and Demographic Survey (SDHS), the country is predominantly rural, with nearly two-thirds of the population residing outside urban centers. Maternal and newborn health services are constrained by geographic, infrastructural, and socioeconomic barriers, contributing to suboptimal utilization of essential maternal and child health interventions.

### Study design

This study employed a cross-sectional design, analyzing nationally representative data from the 2020 SDHS. The survey is part of the global Demographic and Health Surveys (DHS) program and provides standardized indicators of maternal, newborn, and child health.

### Source of data

The analysis used the children recode (KR) file of the 2020 SDHS. The KR file contains detailed information on women of reproductive age (15–49 years) and their most recent live births within the five years preceding the survey. For this study, only the most recent birth per mother was considered, ensuring comparability across cases.

### Sampling and study population

The SDHS 2020 used a two-stage stratified cluster sampling technique. In the first stage, enumeration areas (EAs) were selected using probability proportional to size. In the second stage, households were systematically sampled within each EA. Women aged 15–49 years who had a live birth within five years prior to the survey were eligible. After excluding cases with missing data on the outcome variables, a weighted sample of 2,314 mothers with a recent live birth was included in the analysis.

### Study variables

The outcome variable of interest was essential newborn care (ENC) after birth. Essential newborn care (ENC) is defined as a strategic approach planned to improve the health of new-born through intervention immediately after birth and during the rest of the postnatal period. In line with WHO recommendations and previous Demographic Health Survey-based studies, five indicators were used to define ENC: examination of the newborn's cord, measurement of the newborn's temperature, counseling of the mother on newborn danger signs, counseling of the mother on breastfeeding, and observation of breastfeeding (12). Each of these indicators was recoded into a binary variable, where a value of “1” indicated that the care item was received and “0” indicated that it was not received or the response was “don't know.” These five indicators were summed to generate a composite ENC score ranging from 0 to 5. For this study, the composite score was further dichotomized into a binary variable, where a score of zero represented missing ENC and a score of one or higher represented received ENC. A cumulative score of zero indicated that the newborn missed all five items (categorized as ‘Missing ENC’), while a score of one or more indicated that the newborn received at least one of the essential care items (categorized as “Received ENC”). “This binary variable served as the primary dependent variable in the analysis”.

The independent variables were categorized into individual-level and community-level factors. Individual-level variables included maternal characteristics such as age, educational attainment, marital status, and number of antenatal care (ANC) visits, as well as household characteristics including sex of the household head, wealth index, family size, place of delivery, media exposure, and perceived size of the child at birth. These factors were selected based on their theoretical and empirical relevance to maternal and newborn health practices.

Community-level variables reflected the broader social and environmental context in which individuals resided. These included region, type of residence (urban or rural), community-level.

Poverty, (defined as the proportion of households in a cluster belonging to the poorest and poorer wealth quintiles). Community-level poverty was a derived variable. Community-level poverty was defined as the proportion of households in the cluster belonging to the poorest wealth quintile. “Clusters were categorized as “High Poverty” if this proportion exceeded the national median”. It was calculated by aggregating the proportion of households falling into the two lowest wealth quintiles (poorest and poorer) within each cluster (enumeration area). Clusters were then categorized as having “high” or “low” poverty based on the median value of these proportions. Community-level education was derived from the proportion of women with no formal education. Clusters with a higher-than-median proportion of uneducated women were classified as “Low Education Communities”. Community access to health facilities was based on the proportion of women who reported that distance to the health facility was a “big problem”. Clusters where this proportion exceeded the median were classified as “Poor Access Communities”. community access to health facilities, perceived distance to health facility (measured by the question: “Is distance to the health facility a big problem for you?”). Community-level media exposure (defined as, listening to the radio, or watching television) Community-level media exposure was generated by aggregating the proportion of women who had exposure to either radio or television. “Clusters above the median proportion were classified as “High Exposure””. Media exposure was measured as a composite variable based on two survey questions regarding the frequency of: (1) listening to the radio, and (2) watching television. Respondents were coded as having “Yes” for media exposure if they reported using at least one of these media sources at least once a week. To construct these community-level variables, individual responses within each cluster (enumeration area) were aggregated, and the resulting proportions were dichotomized into “high” and “low” categories using the median value as the cutoff. This approach allowed for capturing contextual influences on newborn care practices beyond individual and household characteristics.

### Data management and statistical analysis

Data were cleaned, recoded, and analyzed using Stata version 17.0 and R statistical software (version 4.3.1). Sampling weights provided by the Demographic and Health Survey (DHS) were applied throughout the analysis to account for the complex survey design and to ensure national representativeness. Descriptive statistics were used to summarize the characteristics of the study population.

Determinants of missing essential newborn care (ENC) were examined using two-level multilevel logistic regression models to account for the hierarchical structure of the data. Individual mothers and their newborns constituted Level 1, while Enumeration Areas (EAs), representing community clusters or primary sampling units, constituted Level 2. A sequential model-building strategy was employed. First, a null (empty) model without predictors was fitted to assess the extent of between-community variation in missing ENC. Individual-level variables were then added (Model 1), followed by community-level variables (Model 2). Finally, a fully adjusted model (Model 3) incorporating both individual- and community-level covariates was fitted while retaining a random intercept to control for clustering effects. Model performance was evaluated using Akaike Information Criterion (AIC), Bayesian Information Criterion (BIC), and log-likelihood values. The Intraclass Correlation Coefficient (ICC) was calculated to quantify the proportion of total variance attributable to between-community differences. Multicollinearity among explanatory variables was assessed using the Variance Inflation Factor (VIF), with all variables showing VIF values below 3, indicating no evidence of multicollinearity.

### Spatial analysis

Spatial analyses were conducted using R statistical software. Spatial data handling, shapefile processing, and geographic data management were performed using the *sf* package. Spatial relationships between geographic units were defined using a queen contiguity spatial weights matrix, which considers regions sharing either a common boundary or vertex as neighbors. This specification was selected to reflect administrative adjacency and to ensure connectivity across all regions. Global spatial autocorrelation in missing essential newborn care was assessed using Global Moran's I statistic implemented through the *spdep* package to determine whether the spatial distribution of missing ENC was clustered, dispersed, or random across Somaliland. Statistical significance was evaluated using standardized z-scores, with conventional confidence thresholds applied.

Local spatial clustering was examined using both Local Moran's I (LISA) and Getis–Ord Gi* statistics to identify localized clusters and hotspot areas. Local Moran's I was used to detect high–high and low–low clusters as well as spatial outliers, while Getis–Ord Gi* statistics were applied to identify areas with significantly high or low concentrations of missing ENC. Given the multiple local comparisons inherent in hotspot analyses, results were interpreted conservatively by emphasizing spatially coherent and consistent clusters rather than isolated statistically significant locations. Spatial visualization and thematic mapping of prevalence, Global Moran's I results, Local Moran's I clusters, and Getis–Ord Gi* hot and cold spots were produced using the *ggplot2* and *tmap* packages. Color schemes were generated using the *RColorBrewer* package to enhance interpretability. Survey-weighted estimates were calculated using the *survey* package, and DHS data were imported using the *haven* package.

## Results

The study provides a detailed snapshot of the demographic and socioeconomic characteristics of 2,314 participants in Somaliland (Table 1). A significant proportion of the study population resides in rural areas (57.7%), highlighting the importance of understanding healthcare access and delivery in these settings. Regional representation was notably higher in Togdheer (28.5%) and Marodijeh (27.9%), while Sahil had the lowest proportion (4.8%). Educational attainment among mothers presents a considerable challenge, with a dominant 76.2% having no formal education, indicating potential barriers to health literacy and engagement with modern healthcare practices. The majority of households are male-headed (61.4%), and an overwhelming 92.0% of mothers are currently married. The age distribution of mothers peaks in the 20–34 age group (67.1%), aligning with typical reproductive years. Economically, the population shows a polarization, with the “rich” category comprising the largest segment (50.8%), followed by the “poor” (37.3%), suggesting significant wealth disparities that could influence access to essential newborn care resources. Furthermore, a substantial 74.2% of participants reported no media exposure, which could limit the reach of public health information and awareness campaigns.

**Table 1:** Descriptive characteristics of study participants (*n* = 2,314).

Variable	Category	Frequency	Percent (%)
Region	Awdal	201	8.7
	Marodijeh	646	27.9
	Sahil	112	4.8
	Togdheer	659	28.5
	Sool	278	12.0
	Sanaag	417	18.0
Type of residence	Rural	1,335	57.7
	Urban	978	42.3
Highest education level	No education	1,763	76.2
	Primary	366	15.8
	Secondary	125	5.4
	Higher	59	2.6
Sex of household head	Male	1,421	61.4
	Female	892	38.6
Current marital status	Married	2,129	92.0
	Divorced	128	5.6
	Widowed	57	2.5
Size of child at birth	Very large	219	9.5
	Larger than average	222	9.6
	Average	1,645	71.1
	Smaller than average	127	5.5
	Very small	101	4.4
Mother's age group	15–19	111	4.8
	20–34	1,552	67.1
	35–49	651	28.1
Wealth status	Poor	862	37.3
	Middle	278	12.0
	Rich	1,174	50.8
Media exposure	No	1,717	74.2
	Yes	597	25.8
Distance to health facility	Not big problem	1,418	61.3
	Big problem	896	38.7
Number of ANC visits	No ANC visit	1,146	49.7
	<4 visits	667	28.9
	4+ visits	493	21.4
Place of delivery	Home	1,336	57.7
	Health facility	978	42.3
Family size	1–5 members	1,174	50.8
	>5 members	1,139	49.2

The data also sheds light on critical aspects of maternal healthcare access and practices. Perceived child size at birth primarily falls within the “average” range (71.1%), with almost equal distributions of “very large” and “larger than average” (9.5% and 9.6% respectively), and “smaller than average” and “very small” (5.5% and 4.4%). Regarding healthcare access, while 61.3% of respondents did not consider distance to a health facility a “big problem”, a significant 38.7% did, pointing to persistent geographical barriers for a considerable portion of the population. Antenatal care (ANC) utilization remains a major concern, as nearly half of the mothers (49.7%) had no ANC visits, and only 21.4% achieved the recommended four or more visits, underscoring a critical gap in preventive care. The place of delivery further emphasizes this challenge, with a majority of births (57.7%) occurring at home rather than in health facilities (42.3%). This preference for home births, often without skilled attendance, has direct implications for the availability and quality of essential newborn care immediately after birth. Family size is fairly evenly split between 1 and 5 members (50.8%) and more than 5 members (49.2%), a factor that can influence household resources and the capacity to provide adequate care for newborns.

Table 2 presents the results from four nested multilevel logistic regression models designed to identify the determinants of missing essential newborn care items after birth in Somaliland. Model 0 is the null model assessing community-level variance. Model 1 includes only individual-level factors. Model 2 includes only community-level factors. Finally, Model 3 is the fully adjusted model combining factors from all levels, which, as confirmed by the model comparison in Table 3 (lowest AIC and, highest Log likelihood), serves as the primary basis for interpretation due to its superior fit and ability to explain both individual and community-level variance.

**Table 2:** Multilevel logistic regression analysis of individual and community-level factors.

Variables	Model 0	Model 1	Model 2	Model 3
		AOR (95% CI) *P*-value	AOR (95% CI) *P*-value	AOR (95% CI) *P*-value
Maternal age				
15–19		1		1
20–34		0.92 (0.49–1.73) 0.791		0.99 (0.52–1.86) 0.958
35–49		1.04 (0.53–2.05) 0.916		1.08 (0.55–2.13) 0.828
Sex of household head				
Male				1
Female		1.04 (0.77–1.41) 0.756		1.08 (0.80–1.46) 0.577
Region				
Awdal		1	1	1
Marodijeh			0.79 (0.46–1.35) 0.386	0.75 (0.44–1.27) 0.296
Sahil			1.03 (0.62–1.71) 0.900	0.95 (0.57–1.58) 0.847
Togdheer			0.18 (0.10–0.33) 0.001	0.28 (0.15–0.51) 0.000
Sool			0.43 (0.26–0.73) 0.001	0.65 (0.38–1.10) 0.115
Sanaag			0.27 (0.16–0.46) 0.001	0.58 (0.33–0.99) 0.047
Residence				
Rural			1	
Urban			1.12 (0.83–1.52) 0.462	1.22 (0.89–1.67) 0.196
Highest educational level				
No education		1		
Primary	–	1.29 (0.87–1.91) 0.208	–	1.41 (0.95–2.09) 0.100
Secondary	–	1.51 (0.78–2.91) 0.224	–	1.47 (0.77–2.81) 0.278
Higher	–	1.47 (0.64–3.38) 0.383	–	1.33 (0.58–3.05) 0.611
Marital status				
Married		1		1
Divorced		0.68 (0.34–1.36) 0.277		0.73 (0.36–1.45) 0.358
Widowed		1.05 (0.42–2.65) 0.919		1.07 (0.43–2.66) 0.911
Family size				
1–5 members				1
More than 5 members		1.15 (0.85–1.54) 0.345		1.12 (0.85–1.53) 0.353
Wealth status				
Poor		1		1
Middle	–	1.72 (1.03–2.89) 0.025	–	2.16 (1.15–4.04) 0.017
Rich	–	1.65 (1.07–2.55) 0.015	–	2.28 (1.27–4.11) 0.008
ANC visit				
No ANC visit				1
<4 visits		1.22 (0.84–1.79) 0.288		1.125 (0.85–1.85) 0.273
4 or more visits		1.36 (0.89–2.09) 0.147		1.37 (0.88–2.11) 0.162
Delivery place				
Home				
Health facility	–	6.75 (4.67–9.75)0.001	–	6.17 (4.24–8.97) 0.001
Size of child				
Very large average		1		1
Larger than average		0.69 (0.39–1.24) 0.221		0.46 (0.29–0.71) 0.313
Average	–	0.42 (0.27–0.65)0.001	–	0.46 (0.30–0.71) 0.001
Smaller than average		1.02 (0.50–2.08) 0.932		1.05 (0.52–2.12) 0.848
Very small		1.32 (0.69–2.55) 0.376		1.31 (0.68–2.50) 0.380
Distance Health Facility				
Not Have big problem		1		1
Big problem		1.04 (0.76–1.43) 0.778	1	1.01 (.074–1.39) 0.962
Media exposure				
No				1
Yes		0.91 (0.62–1.35) 0.669		0.82 (0.55–1.22) 0.339
Community poverty level				
Low poverty			1	1
High poverty			0.24 (0.12–0.45)0.001	1.33 (0.64–2.78) 0.447
Community access health facility				
High access				1
Poor access			1.04 (0.63–1.71) 0.816	1.02 (0.67–1.56) 0.906
Community Media Level				
Low Exposure Community		1	1	1
High Exposure Community			1.45 (0.81–2.59) 0.205	1.13 (0.69–1.87) 0.609
Community education level				
High community education			1	1
Low community education			0.74 (0.44–1.24) 0.279	0.61 (0.40–0.93) 0.021

**Table 3:** Model comparison and random effect analysis result.

Parameters	Model 0	Model I	Model II	Model III
AIC	1,593.687	1,351.199	1,490.901	1,340.08
BIC	1,605.098	1,476.675	1,559.364	1,522.591
Log likelihood	−794.84369	−653.59928	−733.45034	−638.0402
ICC	.2855896	.0885246	.0793409	.0115281
Variance	1.315143	.3195198	.2835156	.0383683

The null Model 0 initially revealed substantial between-community variability with an Intraclass Correlation Coefficient (ICC) of 0.2855896 (Table 3), attributing nearly 29% of the variance to community differences. Table 2 shows that in Model 1, which assessed individual-level factors, delivery in a health facility was a dominant predictor (AOR = 6.75; 95% CI: 4.67–9.75), alongside significant associations for middle wealth status (AOR = 1.72; 95% CI: 1.03–2.89), rich wealth status (AOR = 1.65; 95% CI: 1.07–2.55), and average child size (AOR = 0.42; 95% CI: 0.27–0.65). Model 2 subsequently introduced community-level factors, where Table 2 highlights significant disparities for the Togdheer region (AOR = 0.18; 95% CI: 0.10–0.33) and high-poverty communities (AOR = 0.24; 95% CI: 0.12–0.45), indicating strong geographical and socioeconomic barriers before adjustment for individual characteristics.

The fully adjusted Model 3, which Table 3 confirms as the most robust based on the lowest AIC and highest Log-likelihood, significantly reduced the ICC to 0.0115281 while identifying key determinants as shown in Table 2. In this final model, demographic factors such as maternal age 35–49 (AOR = 1.08), female-headed households (AOR = 1.08), primary education (AOR = 1.41), marital status, and family size were not statistically significant. However, place of delivery remained the strongest predictor, with health facility births showing significantly higher odds of receiving care (AOR = 6.17; 95% CI: 4.24–8.97). Table 2 also indicates that wealth effects intensified in Model 3 for middle (AOR = 2.16; 95% CI: 1.15–4.04) and rich households (AOR = 2.28; 95% CI: 1.27–4.11), and average child size remained significant (AOR = 0.46; 95% CI: 0.30–0.71). Conversely, Table 2 reveals persistent community-level barriers, where mothers in Togdheer (AOR = 0.28; 95% CI: 0.15–0.51) and Sanaag (AOR = 0.58; 95% CI: 0.33–0.99), as well as those in communities with low education levels (AOR = 0.61; 95% CI: 0.40–0.93), were significantly less likely to receive essential newborn care.

Table 3 provides critical insights into the fit and structure of the multilevel models used in the analysis. The comparison across the four nested models—Model 0 (null), Model 1 (individual-level), Model 2 (community-level), and Model 3 (fully adjusted)—demonstrates a progressive improvement in model fit. Model 3 consistently shows the lowest AIC (1,340.08) the highest Log likelihood (−638.0402). Although Model 1 presented the lowest BIC (1,476.675) due to the penalty for increased model complexity, Model 3 was selected as the final model because it exhibited the lowest AIC and allowed for the simultaneous analysis of individual and community-level determinants”. as well as the highest Log likelihood (−638.0402) indicating a significantly better fit to the observed data. Furthermore, the Proportional Change in Variance (PCV) confirms the importance of the fully adjusted model. Model 3 reduced the community-level variance to 0.0383, compared to 0.3195 in Model 1.These metrics collectively indicate that Model 3 is the best-fitting model, successfully capturing more of the variability in the outcome while balancing complexity. Furthermore, The Intra class Correlation Coefficient (ICC), which represents the proportion of the total observed variation (comprising both within- and between-group variance) that is attributable to differences between communities, significantly decreases with each successive model. Starting with an ICC of 0.2855896 in the null model (Model 0), it reduces to 0.0885246 in Model 1, 0.0793409 in Model 2, and finally to a minimal 0.0115281 in the fully adjusted Model 3. This substantial reduction in ICC suggests that the compositional characteristics of the individuals (fixed effects) and the contextual factors included in the models effectively explain the majority of the variation that was initially attributed to differences between communities.

**Figure 1:**
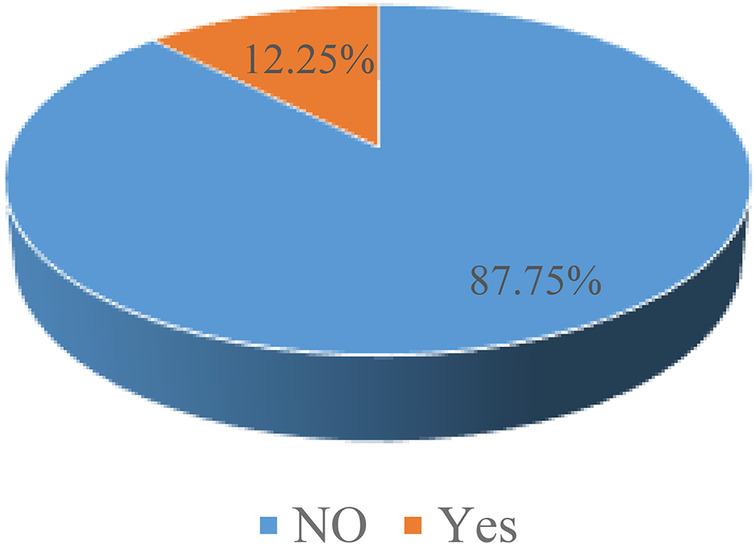
Prevalence of missing essential new born care.

As shown above in Figure 1, the analysis reveals a high burden of missing care. This showed the majority of the study participants, 87.75%, were classified as having “Missing Essential Newborn Care” (indicated as “NO”). Only a small minority, 12.25%, were reported to have successfully received Essential Newborn Care.

**Figure 2:**
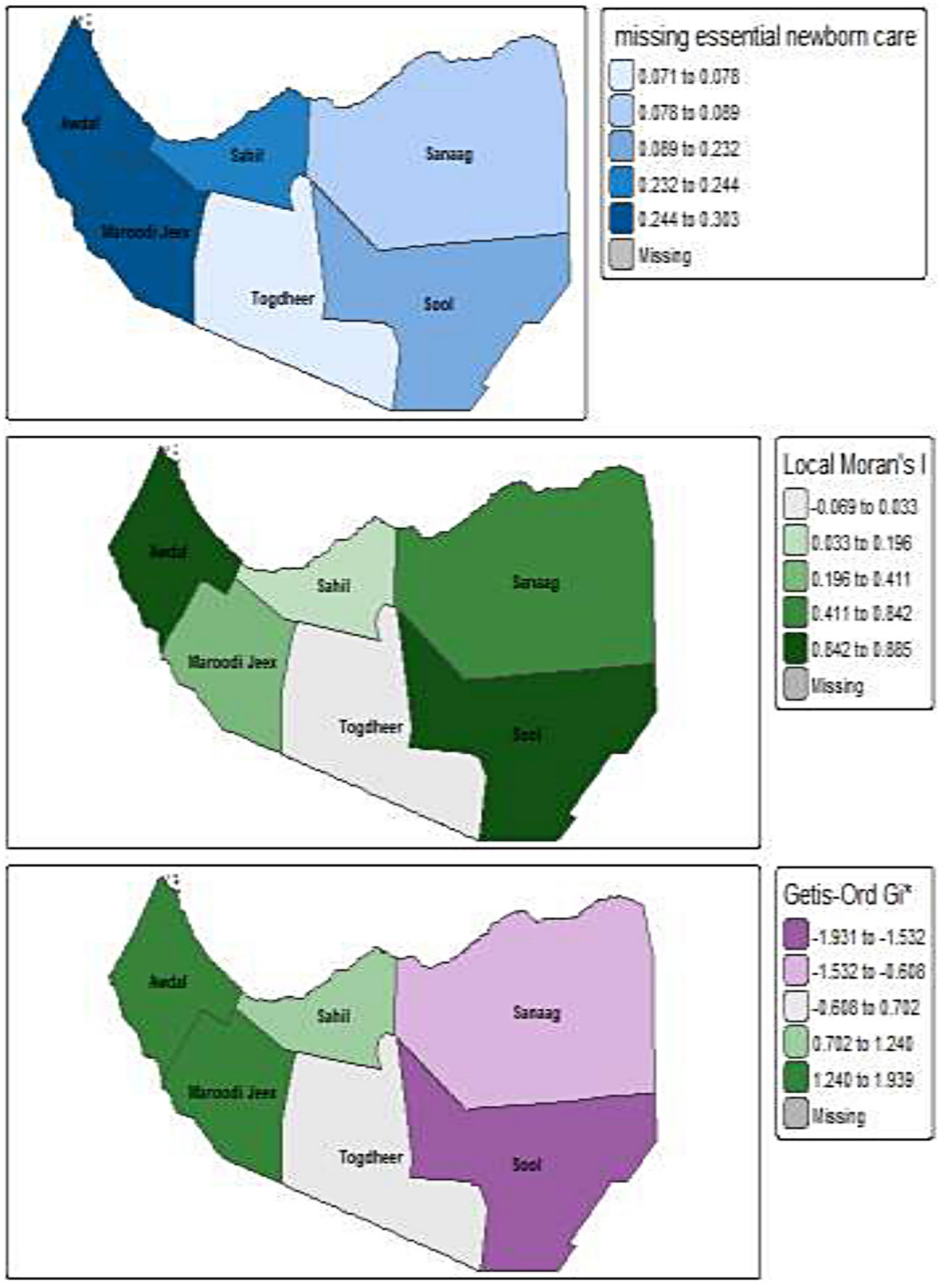
Spatial autocorrelation distribution of missing essential newborn care items after birth in Somaliland.

Figure 2 presents a comprehensive spatial analysis of missing essential newborn care items in Somaliland, comprising three distinct maps that illustrate prevalence, spatial autocorrelation, and significant clustering. Figure 2A (Prevalence map) shows the regional distribution of essential newborn care receipt, where the color gradient highlights a clear geographical disparity. Western regions, specifically Awdal and Marodijeh, appear in darker blue hues, representing the highest proportions of receiving care (approximately 30%) and consequently the lowest prevalence of missing items. In contrast, the eastern regions of Togdheer, Sool, and Sanaag are rendered in lighter blue hues, indicating the lowest proportions of receiving care (as low as 7%) and the most critical gaps in service provision. Figure 2B (Local Moran's I) illustrates the spatial autocorrelation of missing essential newborn care, determining that these patterns are not random. The presence of green shades indicates positive clustering, where regions with similar performance levels are grouped; specifically, the darker green areas in the west signify a strong clustering of high-performing regions, while the lighter green areas in the east suggest weaker clustering among the lower-performing localities.

Figure 2C (Getis–Ord Gi*) statistic to confirm the statistical significance of these clusters based on Z-scores and *p*-values. This analysis identifies Awdal and Marodijeh as significant “Hot Spots” of received care (Z-score > 1.96, *P* < 0.05), representing a statistically significant cluster of regions with a low prevalence of missing essential newborn care. Conversely, Togdheer and Sanaag emerge as significant “Cold Spots” (Z-score < −1.96, *P* < 0.05), signifying a significant clustering of low service coverage and a high prevalence of missing care. The distinction between the “Hot Spots” (often represented in purple for high missing care in the initial description, though the statistical conclusion emphasizes the “Hot Spots” of received care in the west) and “Cold Spots” quantitatively confirms the observations from Map A. Ultimately, this comprehensive spatial analysis demonstrates that the absence of essential newborn care is structurally entrenched, characterized by significant geographical barriers that disadvantage the eastern regions compared to the west.

**Figure 3:**
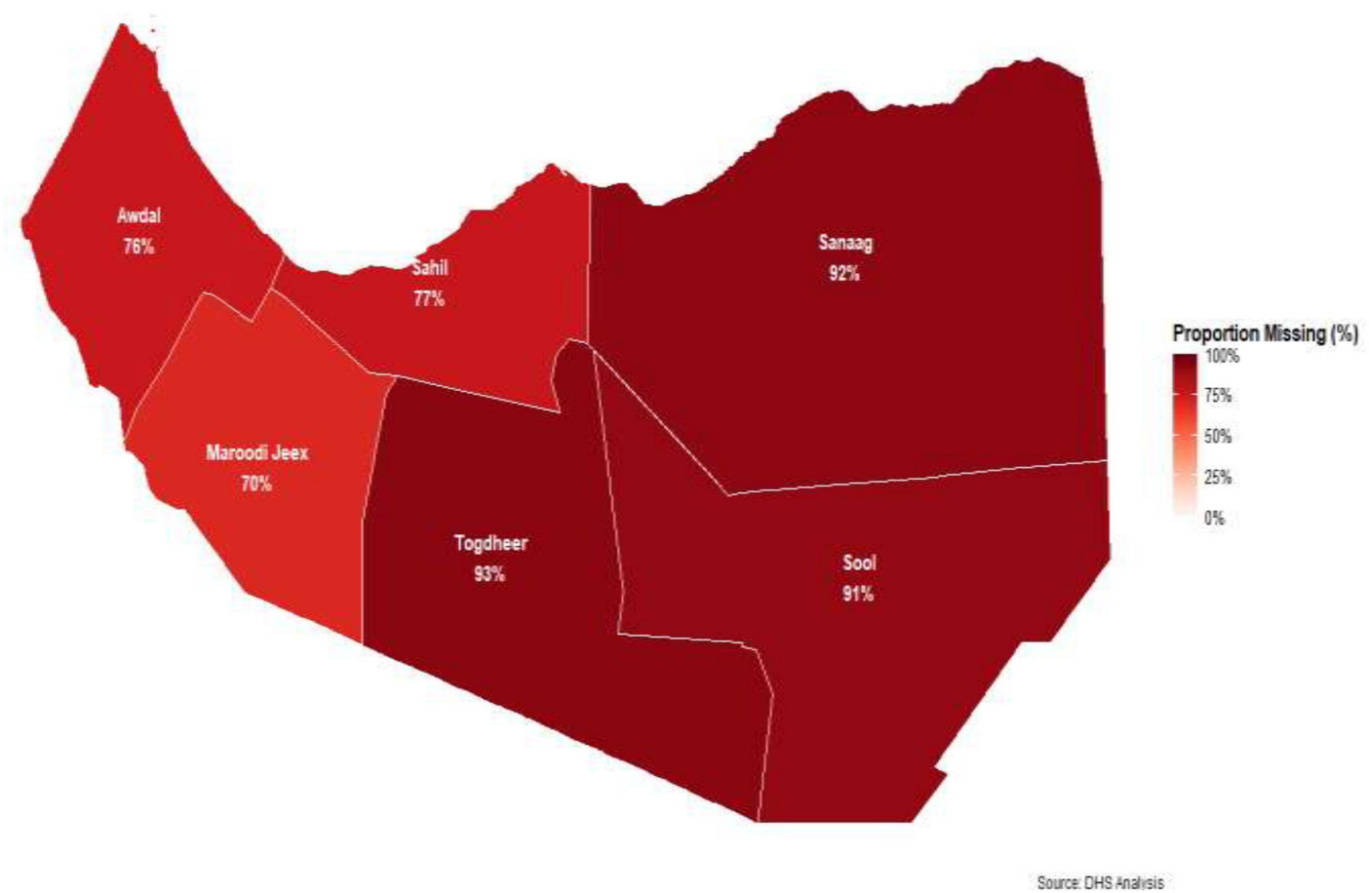
Spatial distribution of missing essential newborn care items after birth in Somaliland.

Figure 3 reveals significant spatial disparities. For instance, Sanaag, Togdheer, and Sool regions show the highest proportions of missing items, at 92%, 93%, and 91% respectively. This suggests a critical need for interventions in these areas. Regions like Awdal (76%), Sahil (77%), and Marodijeh (70%) also demonstrate high proportions of missing items, though slightly lower than the aforementioned regions. The overall high percentages across all regions, with none falling below 70%, underscore a widespread challenge in access to essential newborn care items throughout Somaliland. This visual evidence of spatial variation will be crucial for your paper in identifying areas requiring targeted interventions and for further investigating the underlying determinants of these disparities through your spatial and multilevel analysis of SDHS data.

**Figure 4:**
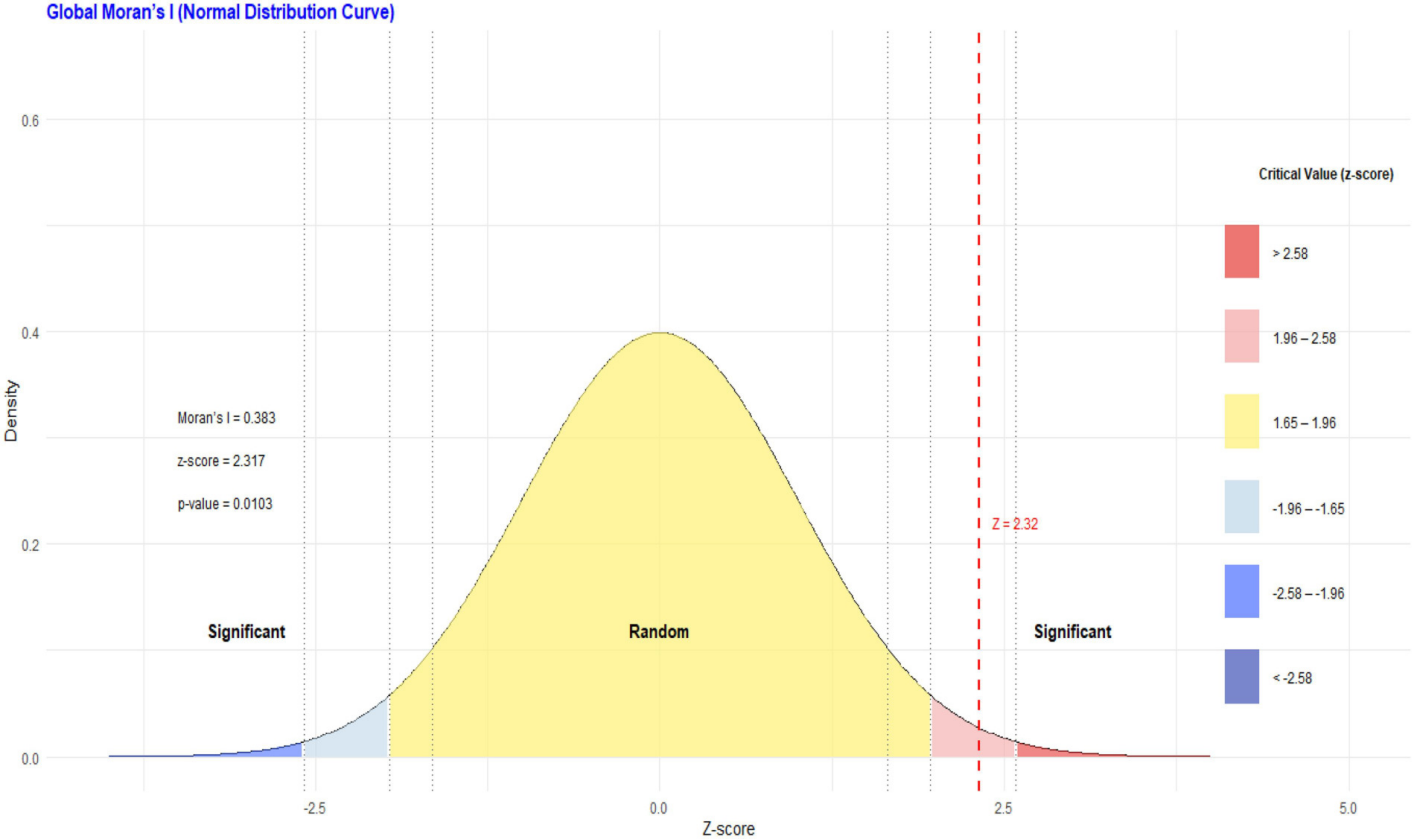
Global Moran's I for the spatial autocorrelation.

Figure 4 illustrates the standardized normal distribution of Moran's I statistic used to assess global spatial autocorrelation. The dashed red line represents the observed z-score (z = 2.32). The right-side panel displays critical z-value thresholds used to determine statistical significance. The results indicate significant positive spatial autocorrelation (Moran's I = 0.38; *P* = 0.0103), demonstrating that missing essential newborn care items are spatially clustered rather than randomly distributed across regions.

## Discussion

This study provides the first spatial and multilevel analysis of missing essential newborn care (ENC) in Somaliland and demonstrates that gaps in ENC are not randomly distributed but are strongly shaped by geographic, socioeconomic, and health system factors. The findings indicate that missing ENC reflects broader structural constraints within the maternal and newborn health system rather than isolated individual behaviors.

The strong association between place of delivery and receipt of essential newborn care highlights the central role of health facilities as the primary platform for delivering ENC services in Somaliland. Facility-based births are more likely to be attended by skilled health personnel who can provide standardized newborn care practices immediately after birth, including thermal protection, early breastfeeding support, and newborn assessment. In contrast, home births—often assisted by traditional birth attendants or family members—frequently lack access to trained providers, essential equipment, and clear clinical protocols. This pattern aligns with evidence from multiple low- and middle-income settings, where home delivery has consistently been associated with lower coverage of essential newborn care practices (21–23). In Somaliland, where home births remain common and skilled birth attendance is limited, these gaps are likely amplified by shortages of health workers, uneven facility readiness, and weak referral systems, particularly in rural and remote areas (19).

Socioeconomic status emerged as an important determinant of ENC utilization, underscoring the persistent influence of economic inequality on access to maternal and newborn health services. Although maternal and newborn care services are officially provided at low or no cost in Somaliland, indirect expenses such as transportation, accommodation near health facilities, and opportunity costs related to household responsibilities continue to pose substantial barriers for poorer families. Wealthier households are better able to absorb these costs and navigate the health system, increasing the likelihood of facility delivery and timely postnatal care. Similar wealth-based disparities in essential newborn care have been documented across low- and middle-income countries, including Ethiopia and other sub-Saharan African settings, indicating that financial disadvantage remains a critical barrier to equitable newborn care utilization (4, 24, 25).

Marked regional disparities in ENC coverage were observed, with eastern regions—particularly Togdheer, Sanaag, and Sool—consistently exhibiting lower levels of essential newborn care compared to western regions such as Awdal and Maroodi Jeex. These geographic differences persist even after accounting for individual and household characteristics, suggesting that structural health system factors play a decisive role. Eastern Somaliland is characterized by sparse health infrastructure, long distances between communities and health facilities, limited availability of skilled health workers, and a higher proportion of nomadic and semi-nomadic populations. These conditions constrain both the supply of and demand for essential newborn care services, reducing opportunities for timely postnatal interventions. Similar patterns of subnational inequities in maternal and newborn health service utilization have been reported in other settings, including India, where remote and underserved regions consistently lag behind more developed areas despite national-level improvements (26, 27).

The association between perceived newborn size and receipt of essential newborn care suggests that caregiver perceptions and provider practices may influence newborn care delivery. Smaller or perceived low-birth-weight newborns may be viewed as fragile, leading to delayed handling or reduced provision of routine care practices, particularly in settings with limited clinical capacity to manage vulnerable infants. Evidence from Ethiopia supports this interpretation, showing that smaller newborns are less likely to receive recommended essential newborn care practices (20). This finding highlights the importance of strengthening provider training and facility readiness to ensure that vulnerable newborns receive appropriate care rather than being unintentionally excluded from routine ENC practices.

At the community level, the finding that lower community education was associated with a lower likelihood of missing ENC was unexpected but may reflect the targeted deployment of maternal and newborn health programs in disadvantaged areas. In Somaliland, NGO-supported interventions and outreach services are often concentrated in underserved communities with lower literacy levels, potentially mitigating the negative effects of low formal education on newborn care practices. This contrasts with findings from Ethiopia and Ghana, where higher community education levels were generally associated with better essential newborn care practices, underscoring the context-specific nature of community-level influences on ENC utilization (28, 29).

Overall, the findings indicate that missing essential newborn care in Somaliland is driven by a combination of health system limitations, geographic inequities, and socioeconomic barriers. The spatial clustering of poor ENC coverage in eastern regions highlights the need for geographically targeted strategies rather than uniform national approaches. While the determinants identified in this study are broadly consistent with evidence from other low- and middle-income countries, the spatial patterns observed emphasize the importance of tailoring newborn health interventions to Somaliland's unique health system constraints and population dynamics.

### Policy implications and recommendations

The findings indicate that improving essential newborn care (ENC) in Somaliland requires geographically targeted and context-specific interventions rather than uniform national approaches. Given the spatial clustering of missing ENC in eastern regions—particularly Togdheer, Sanaag, and Sool—these areas should be prioritized for intensified maternal and newborn health actions.

The Ministry of Health should focus on strengthening the readiness of existing health facilities in hotspot districts, ensuring the availability of skilled birth attendants, essential newborn care supplies, and basic referral capacity. To address the high prevalence of home births, community-based postnatal outreach, including early home visits by trained community health workers within the first 48 h after birth, should be expanded, especially in remote and nomadic communities.

Socioeconomic barriers identified in this study suggest the need for pro-poor access mechanisms, such as transport support or community-managed referral funds, to reduce indirect costs that limit access to facility delivery and postnatal care among poorer households. In addition, targeted training for facility and community health workers should emphasize the care of small or vulnerable newborns to ensure consistent delivery of essential newborn care practices.

Finally, integrating subnational monitoring and spatial evidence into routine planning and partner coordination will support more equitable allocation of resources and improve newborn health outcomes across Somaliland.

## Conclusion

This study presents a comprehensive spatial and multilevel analysis of essential newborn care (ENC) in Somaliland, miss vital care practices, a deficit primarily driven by geographic clustering and systemic barriers. Spatial analysis revealed significant non-random variation, identifying statistically substantial hotspots of missing essential newborn care in the eastern regions of Sanaag, Sool, and Togdheer. In contrast, the western regions of Awdal and Maroodi Jeex emerged as cold spots, reflecting relatively better care coverage. Multilevel modeling further demonstrated that place of delivery is the strongest determinant of ENC utilization, with facility-based deliveries markedly increasing the likelihood of receiving care compared with home births. Persistent socioeconomic inequities were also evident, as newborns from wealthier households had significantly higher odds of receiving ENC, whereas those residing in eastern regions and communities with lower educational attainment faced substantially reduced access. Collectively, these findings highlight the urgent need for geographically targeted interventions in high-risk eastern regions, alongside broader strategies to promote facility-based deliveries and address socioeconomic disparities to improve neonatal survival across Somaliland.

## Limitation

This limitation was, first, the cross-sectional design captures data at a single point in time, limiting the ability to establish temporal relationships or causality between determinants and missed essential newborn care (ENC). Second, the analysis relies on maternal recall from the 2020 Somaliland Demographic and Health Survey, with births reported up to five years before the survey, introducing potential recall bias, particularly for specific ENC components. Third, birth size was measured using maternal perception rather than objective birth weight, which may be influenced by subjective norms and lead to misclassification bias in assessing its association with ENC receipt.

## Data Availability

The data used in this study were obtained from the Central Statistics Department, Ministry of Planning, Somaliland. The dataset is not publicly available due to institutional restrictions but can be accessed upon reasonable request by contacting the Central Statistics Department, Ministry of Planning, Somaliland. approval and consent to participate. Requests to access these datasets should be directed to admin@somalilandcsd.org.
