# Supplementary material for: Spatial distribution and determinants of missing essential newborn care items after birth in Somaliland: a spatial and multilevel analysis of SDHS
Source: Front Pediatr. 2026 Feb 6;14:1732341. doi: 10.3389/fped.2026.1732341 (PMC12920529; doi:10.3389/fped.2026.1732341)
Supplement: Supplementary file 1 [file Datasheet1.pdf]

Multicollinearity Test

Variable	VIF	1/VIF
**Model 1 Individual factors**		
Wealth status	1.55	0.646491
Place of delivery	1.53	0.655681
Number of ANC visits	1.42	0.703852
media exposure	1.28	0.778901
Highest education level	1.25	0.799042
Distance to health facility	1.11	0.899851
family size	1.02	0.983327
Size of child at birth	1.02	0.984378
Sex of household head	1.02	0.984708
Current marital status	1.01	0.990402
Mean VIF	1.22	
**Model 2 Community factors**		
Community poverty level	2.04	0.490769
Community Media Level	1.97	0.508298
Community education level	1.62	0.619102
Community access health facility	1.50	0.665358
Residence	1.00	0.996467
**Model 3 Individual and community factors**		
Wealth status	2.78	0.359486
Place of delivery	1.61	0.622327
Number of ANC visits	1.52	0.656407
media exposure	1.37	0.729881
Highest education level	1.29	0.775841
Distance to health facility	1.17	0.856316
family size	1.02	0.981051
Size of child at birth	1.02	0.981939
Sex of household head	1.02	0.976374
Current marital status	1.02	0.980166
Community poverty level	2.28	0.437793
Community Media Level	2.28	0.438380
Community education level	2.10	0.476723
Community access health facility	1.71	0.585199
